# Our Experience With VEMFtherapy on Skin Aging of Face and Neck

**DOI:** 10.1111/jocd.70778

**Published:** 2026-07-19

**Authors:** Lombardo Fabrizio, Scacciati Chiara, Fabris Camilla, Menchini Giovanni

**Affiliations:** ^1^ Dermatology, Plastic Surgery and Aesthetic Medicine Istituto Dermacademy Pisa Italy

**Keywords:** aging, Biodermogenesi, electromagnetic field, senescence, skin, vacuum, VEMFtherapy, wrinkle

## Abstract

**Introduction:**

In a civil society with increasing life expectancy and average age, aging, including skin aging, is an increasingly important issue. Attenuating skin senescence improves both our appearance and quality of life, promoting social relations.

**Materials and Methods:**

We selected and treated 28 patients, mostly women, aged between 30 and 78 year, subjecting them to a cycle of 6 sessions on a weekly basis of VEMFtherapy, also known as Biodermogenesi. The results were evaluated using a professional camera and 3D detection system such as LifeViz Quantify and Visia. We adopted two scales for wrinkle assessment: the “Wrinkle Assessment Scale” and a scale we use within our clinic. To complete the assessment, we used the Face‐Q questionnaire.

**Results:**

All the assessment methods adopted, both subjective and objective, showed a significant reduction in the evidence of wrinkles as well as the total absence of side effects and downtime. Patients define the therapy as pleasant and relaxing.

**Conclusions:**

Considering the evidence of improvement obtained, the total absence of side effects and downtime and the high level of comfort expressed by patients, it is considered that VEMFtherapy can become an important therapeutic option for skin aging.

## 1 Introduction

### 1.1 Epidemiology of Skin Aging

One of the challenges of the XXI century is the fight against aging, defined as a set of physiological mechanisms that alter the physical and intellectual capacities of human beings [1]. The global population of over‐sixties is increasing at a faster rate than the younger age groups. This trend started in high‐income countries and is now taking place everywhere else [2].

Although in some cultures age is associated with wisdom and respect, in western societies it is mainly a source of concern because of related pathologies such as cardiovascular diseases, type 2 diabetes, dementia, cancer and chronic and degenerative inflammatory processes. Aging‐related chronic diseases affect almost all organs, including the skin, and promote multi‐morbidity that burden health services and society in general, with important economic consequences [3, 4]. The human body comprises highly complex structures, networks of tissues, organs, proteins and genes whose homeostatic balance deteriorates during aging, and the skin, body's most extensive organ, is constantly exposed to endogenous and exogenous stimuli that affect its morphology and function, representing the visible and aesthetic part of this process [1, 4, 5]. The aim of this study is to evaluate the efficacy and safety of VEMFtherapy in the treatment of skin aging.

### 1.2 Physiopathology of Skin Aging—Intrinsic and Extrinsic

Skin aging is a complex process resulting from numerous biological, biochemical and physical interactions that ultimately lead to damage and alteration of its functions involving all the different layers of the skin and supporting tissues.

As the years pass, the dermo‐epidermal joints are weakened, the dermis atrophies and a reduction of cellularity, vascularization and extracellular matrix (ECM) is observed [1]. The changes and deterioration of skin functions begin very early with a progressive morphological and physiological decline that can be classified as exogenous or endogenous aging [6]. Endogenous skin aging is an inevitable process; a succession of chronological and physiological alterations [5]. Endogenously aged skin reflects the degradation processes of the whole organism [6], including genetic ones, such as mutations, which can promote certain diseases or neoplastic processes, genomic instability and genomic polymorphisms [1, 2, 3, 4, 5]. Hormones and gender‐specific factors can also play an important role in skin morphology and the endogenous aging process. Men's skin has a thicker dermis than women's, but it has a thinner hypodermis, suggesting the influence of sex hormones [7]. Cellular aging was first described by Hayflick and Moorhead, who showed that human primary fibroblasts have a limited ability to divide. This phenomenon, known as the Hayflick limit, results from the inability of telomeres to maintain their length due to the replication process, inhibiting in turn their proliferative capacity by entering a state of irreversible cell cycle arrest, defined cellular senescence [5, 8]. Senescent cells are characterized by the inability to proliferate, the resistance to apoptosis and the secretion of factors that promote inflammation and tissue deterioration. Intrinsic aging is also an oxidative process linked to a progressive decrease in antioxidant capacity and increased production of reactive oxygen species (ROS). The clinical signs that correspond to intrinsic skin aging are fine lines, xerosis (dry skin) and laxity [5]. Finally, the decrease and rarefaction of the content of collagen in the skin plays an important role in the aging process. The total collagen content per skin surface unit is known to decrease by about 1% per skin surface unit per year [6]. In older skin, collagen also has an irregular and disorganized appearance, and it has been shown that the ratio between collagen I and collagen III increases mainly due to the loss of collagen type III. As an organ that works as a barrier, the skin also undergoes the so‐called extrinsic aging, linked to the multiple environmental factors to which it is exposed and that today are identified with the term “exposome” [1], which describes the overall exposure of an individual, from birth to death, and includes external and internal factors such as sunlight, air pollution, cigarette smoke, nutritional factors, temperature, stress and lack of sleep and the response of the human body to these factors [9]. Extrinsic aging is mainly limited to exposed areas of the human body, such as face, neck and hands; it overlaps with the intrinsic one depending on intensity, duration of exposure to environmental factors and skin type, and manifests itself mostly in marked wrinkles, uneven pigmentation and freckles or age spots. The most significant extrinsic aging factor is still UV radiation [5]. These factors depend on individual behavior and may be targeted for aging prevention [1].

### 1.3 Social Impact of Aging

It is estimated that, by 2050, all regions of the world with the exception of Africa, will have at least a quarter of the population over 60 years of age, therefore, anti‐aging medicine will become even more important [2, 10]. The process of aging is a complex phenomenon that affects the psychological and social sphere of individuals, and is often treated as a secondary phenomenon to biological aging; however, changes occurring due to aging affect mood, attitude towards the environment, physical condition and social activity, especially in a modern society that promotes youth, progress, development and efficiency [11]. As a result, over the past 20 years there has been an increase in the number of medical aesthetic procedures worldwide, combining the concept of “health” with that of “beauty” [12]. Skin aging is the most visible physical and psychological aspect of aging, but this also makes it the most targeted by both prevention and rejuvenation [13].

### 1.4 Facial Aging and the Role of Gaze

Appearance plays an important role in perceiving oneself and others and defining one's personal well‐being and no other part of the body plays such an important role in this matter as the face [14]. The face is particularly affected since it suffers from exogenous effects, but also from the redistribution of superficial and deep fat and muscle contractions related to facial expression. All these phenomena lead to changes in the skin and shape of the face and its outline [15]. Of all these changes, those of the periorbital area are among the first and the most important to arise and cannot be underestimated, because in this area even small changes of structure or volume can have an effect on an individual's perception of emotions, energy and health [14, 15, 16]. With the aging of the periocular region, patients experience a major eyelid laxity caused by eyelid thinning, lack of lower eyelid support due to loss of cheek volume and the appearance of descending eyebrows due to resorption of the orbital bone that makes the eyes look more recessed in the orbit. To ensure a safe and natural‐looking rejuvenation of the eyelids and supporting structures, an advanced knowledge of anatomy ideal facial proportions and the most effective methods for rejuvenation of this area is required, which is particularly challenging due to the complex and delicate anatomy of the periocular area [16].

## 2 Materials and Methods

The study involved 28 patients, 1 man and 27 women aged between 30 and 78 years, with an average age of 50.75 and a median age of 52, all with varying grades of aging who underwent a facial and neck rejuvenation protocol with VEMFtherapy at our institute between February 2022 and December 2023. The protocol provides one session per week for a minimum of 6 sessions; 7 patients also underwent the periocular area treatment with the SPECIAL handpiece. For this study, we acquired photographs and images of the patients to be evaluated in different ways and equipment that also allowed us to extrapolate specific data on the severity of the blemish. We adopted a “Wrinkle Assessment Scale” [17] and another one created our own, simple and intuitive one, called WES (Wrinkle Evaluation Scale), and evaluated the outcomes with both scales. Finally, we asked patients for their subjective assessment of the results using the Face‐Q questionnaire. The “Wrinkle Assessment Scale” involves the evaluation of 10 specific wrinkles on the face with 6 levels of evidence from 0 (no evidence) to 6 (extremely deep wrinkles), while our WES examines three‐thirds of the face and expresses an overall level of evidence of the wrinkles present in that area with 5 levels starting from 0 (no evidence) to 5 (many wrinkles and extremely deep wrinkles).

The study was conducted in accordance with the Helsinki Declaration of 1975 and its amendments of 1983 and MEDDEV 2.7.1, 4th edition. The results were evaluated using the Wilcoxon Signed Rank, which compares the final data with those obtained at the beginning of this study; P less than 0.05 was considered significant. All patients signed informed consent and authorized the use of their photographs before starting treatment, authorizing the use of their data for this publication.

### 2.1 Method of Image Acquisition

Digital images of the patients' faces were acquired with three different technologies, at the beginning of the treatment and at each subsequent session. The photographs were taken with the camera Canon Eos 4000D in the following projections: frontal, ¾ right, ¾ left, right profile and left profile; the shots were always taken in the same environment and under the same lighting. 3D images of the face were also taken with LifeViz Quantificare camera, then reconstructed with a special program. Finally, digital images of the patients were taken with Visia equipment set to acquire these module positions: Left 33°, Centre 0°, Right 33°. The Visia system has been successfully validated in providing objective data for clinical follow‐up studies on various aspects of the skin [18, 19, 20, 21]. For our study, we used a filter designated for wrinkles that is able to highlight both deep (dark green) and fine (light green) wrinkles of the periocular region, visible in the right and left profiles of each patient. The absolute score, which is what we have chosen to use in our study, provides a precise measurement (size, area, intensity of the blemish) of the parameter of interest to be measured on the skin, wrinkles in our specific case, therefore, it can be necessary to monitor the progress of a treatment in time regarding the considered parameter [18].

### 2.2 Classification of Wrinkles

Regarding the classification of wrinkles, we decided to adopt the “Wrinkle Assessment Scale”, created by Lamperle et al. [17] and another one developed by us which considers the entire aspect of the wrinkle and its appearance. The choice derives from the fact that there is no universally recognized scale [17] for the evaluation of wrinkles, therefore, we adopted a double evaluation method, adding to the “Wrinkle Assessment Scale” our simple and intuitive scale based on the photographic image of the wrinkles analyzed in three thirds of the face, setting a score from 1 to 5, increasing in relation to the severity of wrinkles (Figure S1).

### 2.3 Face‐Q Scale

Measuring quality of life through questionnaires is a common method of assessing the impact of different pathologies on patient well‐being, especially in dermatology where the diseases that change the appearance are common [22]. In our study, we decided to evaluate the impact of aging on patients' lives and their satisfaction rate relating the treatment through Face‐Q [23, 24, 25] questionnaire qualified by the U.S. FDA as a medical device development tool (MDDT). There are three scales belonging to the domain “health‐related quality of life” which work independently and which we decided to select in our study, based on our aims and adequacy with the protocol under review:

(1) The Satisfaction with Facial Appearance Scale.

(2) The Psychological Well‐Being Scale.

(3) The Visual Analog Scale for assessing facial appearance.

Patients were asked to complete these 3 FACE‐Q scales at baseline (day 1) and at the end of the treatment protocol. Regarding aesthetic medical procedures focusing on the face, patient satisfaction with their appearance and overall quality of life (QOL) improvement are perhaps the most important outcome.

### 2.4 System Combining Supply of Electromagnetic Fields, Square Wave Stimulation and Vacuum, and Specific Treatment Program

VEMFtherapy, also known as Biodermogenesi, is delivered by BI‐ONE LifeTouchTherapy device (Expo Italia srl, Firenze, Italia) intended for use in dermatological treatments of scars and stretch marks with excellent results [26]; its effectiveness is also proven in overall facial rejuvenation [27]. The device supplies negative pressure from 9 to 16 mbar, an electromagnetic field with frequency ranging from 0.5 to 2 MHz with average output power of 4 W at 500 Ohm and a 5 Hz square wave with a maximum intensity of 3.5 VPP at 500 Ohm.

The device is equipped with an AI system that independently adjusts output frequency and intensity according to the biological features of the skin of the patient ensuring maximum yield in the absence of side effects.

The three energy forms used are well‐known and have extensive bibliographic documentation. Their simultaneous application allows for effective synergy. Square wave electrical stimulation allows for the absorption of cosmetics and drugs through the stratum corneum [28]. The electromagnetic field is able to influence the activity of sodium and potassium ions across cell membranes, and more precisely through electropores [29, 30]. The generation of a negatively charged electromagnetic field pushes Na + and K+ towards the inside of cell membranes, where the external lipid layer constitutes an electrical and electromagnetic insulator. By varying the polarity, we will instead have an action of equilibrium of the charges, which will tend to reposition themselves in the extracellular matrix. The continuous variation of polarity allows for a double result: the nourishment of the cells and their detoxification. The continuous movement of N+/K+ ions through cell membranes, as a result of friction, is partially transformed into thermal energy, based on what is established by the second law of thermodynamics [31]. Artificial intelligence therefore allows a stabilization of the temperature of the dermis between 39°C and 40°C, thus preventing damage resulting from excessive thermalization [32]. At the same time, the vacuum stimulates the muscles by promoting alternating vasodilation and vasoconstriction generated by the sliding of the handpiece. This effect is amplified by the increase in skin temperature, based on the Arrhenius equation [33], which states that by heating fluids, they reduce their density and viscosity, increasing their flow within the capillaries and consequently proportionally increasing the exchange with the matrix. Similarly, the lower density of the lymph reduces the internal pressure of the vessels and, together with the increase in caliber caused by the negative pressure, activates osmosis to the maximum level, recovering greater volumes of toxins from the extracellular matrix [34, 35]. These actions led to a real regeneration, as demonstrated by Scarano et al. [36], where histochemical analysis highlighted the production of type III collagen, elastic fibers and angiogenesis.

Each session takes between 20 and 25 min; the exclusion criteria are as follows: Patients with pacemaker, epileptics, patients with a history of oncological treatment or surgery in the previous 5 years, pregnant or breastfeeding women, patients with damaged, inflamed and/or sensitized facial and neck skin, suffering from edema or hematomas or active acne. According to the literature, the only potential risk is linked to the formation of petechiae, which are normally reabsorbed within 1 or 2 days [27, 36, 37, 38]. The impressions of patients were collected during all treatment sessions (uncomfortable feeling, discomfort, pain).

## 3 Results

The treatment proved to be effective, pleasant, relaxing and safe. No patient complained of discomfort or pain during and after the therapies and only in one session petechiae occurred, but reabsorbed throughout a day.

### 3.1 Grades of Wrinkles

#### 3.1.1 WES (Wrinkle Evaluation Scale)

Using the WES (Wrinkle Evaluation Scale) classification scale for wrinkles, at the beginning of the treatment protocol (T0) the group of subjects presented as shown in Table 1.

**TABLE 1:** Measurements taken at T0, before the sessions.

T0—Before treatment cycle	Grade 1	Grade 2	Grade 3	Grade 4	Grade 5	Average
Upper third of the face	1	6	13	4	4	3,14
Middle third of the face	0	6	9	9	4	3,39
Lower third of the face	0	7	5	12	4	3,46

At the end of the treatment protocol (T1), the grades of the wrinkles modified as shown in Table 2.

**TABLE 2:** Measurements taken at T1, after a cycle of treatment sessions.

T1—After the treatment cycle	Grade 1	Grade 2	Grade 3	Grade 4	Grade 5	Average
Upper third of the face	1	15	3	4	0	2,43
Middle third of the face	3	14	8	3	0	2,39
Lower third of the face	6	10	11	1	0	2,25

*Note:* The mean level of wrinkle evidence in the three areas analyzed according to the WES (Wrinkle Evaluation Scale) was 3,33 at T0 and reduced to 2,36 at T1, with an average improvement of 29,13%.

#### 3.1.2 Wrinkle Assessment Scale

Using the Wrinkle Assesment Scale for wrinkles, at the beginning of the treatment protocol (T0) the group of subjects presented as shown in Table 3.

**TABLE 3:** Measurements taken at T0, before the sessions.

T0—Before treatment cycle	Grade 0	Grade 1	Grade 2	Grade 3	Grade 4	Grade 5	Average
Horizontal Forehead Lines	0	4	5	7	6	6	3.18
Glabellar Frowns	1	4	4	8	7	4	3.00
Periorbital Lines	0	4	6	7	6	5	3.07
Preauricular Lines	1	5	4	7	6	5	2.93
Cheek Folds	0	4	5	8	6	5	3.11
Nasolabial Folds	0	3	7	8	6	4	3.06
Upper Lip Lines	1	3	5	7	7	5	3.11
Corner of Mouth Lines	0	3	5	8	8	4	3.18
Marionette Lines	0	3	5	9	6	5	3.18
Chin Crease	0	4	6	7	6	5	3.07

At the end of the treatment protocol (T1), the grades of the wrinkles modified as shown in Table 4.

**TABLE 4:** Measurements taken at T1, after a cycle of treatment sessions.

T1– After treatment cycle	Grade 0	Grade 1	Grade 2	Grade 3	Grade 4	Grade 5	Average
Horizontal Forehead Lines	1	7	12	4	4	0	2.11
Glabellar Frowns	2	6	8	7	5	0	2.36
Periorbital Lines	1	7	9	6	5	0	2.25
Preauricular Lines	2	7	7	7	5	0	2.21
Cheek Folds	2	7	7	7	5	0	2.21
Nasolabial Folds	2	6	8	7	4	1	2.29
Upper Lip Lines	2	6	8	7	5	0	2.25
Corner of Mouth Lines	2	6	9	7	3	1	2.14
Marionette Lines	3	5	8	6	5	1	2.29
Chin Crease	2	5	9	5	4	2	2.29

*Note:* The mean level of evidence for the ten main wrinkles analyzed according to the “Wrinkle Assessment Scale” was 3.09 at T0 and reduced to 2.24 at T1, with a mean improvement of 27.51%.

#### 3.1.3 FACE‐ Q Assessment

Assessment of the three scales of FACE‐Q reported the following outcomes (Table 5).

**TABLE 5:** FACE‐Q patients' subjective assessment.

Examined data	T0	T1	Difference
Satisfaction with Facial Appearance	22,96	26,82	+3,86/ +16,81%
Psychological Well‐Being	21,75	25,57	+3,82/ +17,56%
Evaluation of the Apparent Age	+3,4 years	+1,36 years	‐2,04 years/‐60%

#### 3.1.4 Assessment of PERIOCULAR WRINKLES With VISIA

Changes recorded with Visia are the following (Table 6).

**TABLE 6:** Measurement with Visia.

Assessment time	Wrinkles severity average value	Wrinkles severity median value
T0 – Before treatment	37,70	38,17
T1 – After treatment	33,80	33,14
Changes	‐3,90/‐10,35%	‐5,03/‐15,17%

## 4 Discussion

This study has documented the effectiveness and safety of VEMFtherapy in face rejuvenation with an improvement in severity of wrinkles located in the three sections of the face, and in absolute scoring parameter with Visia system and FACE‐Q scale.

**FIGURE 1:**
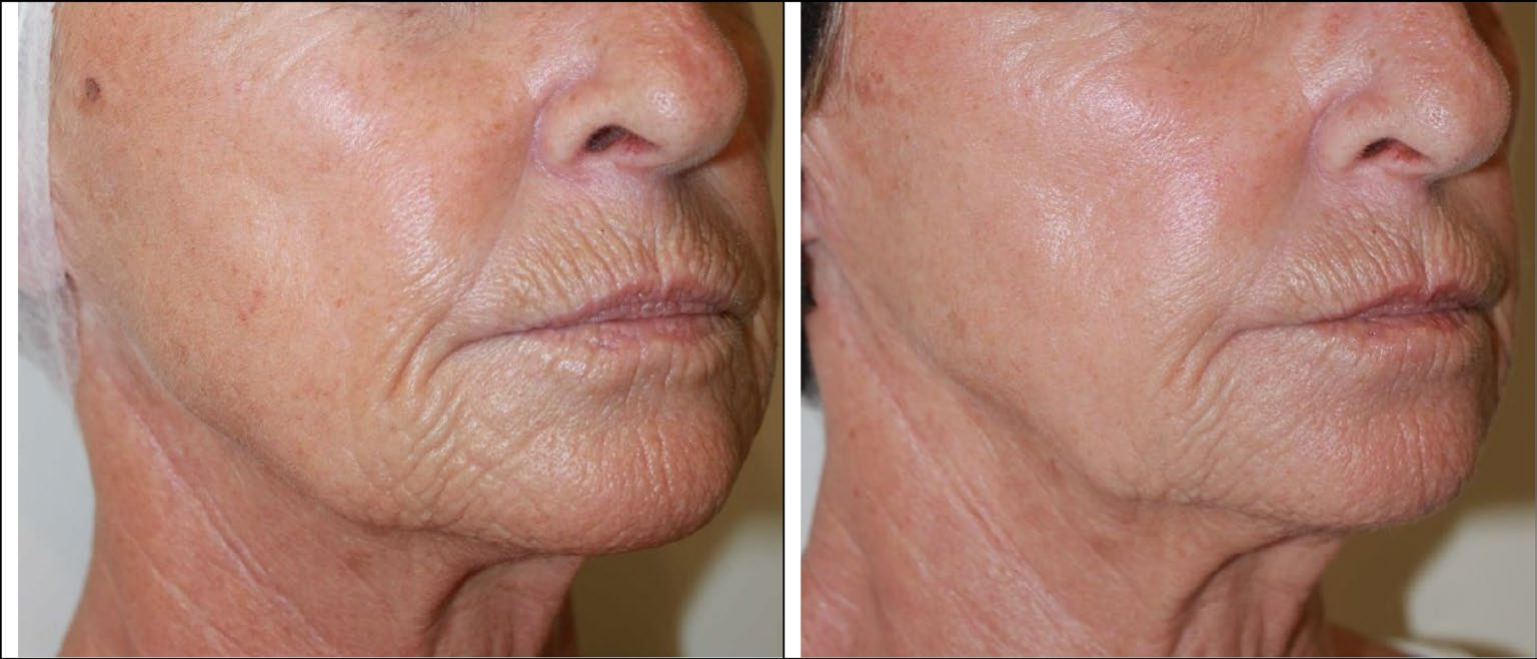
A reduction of wrinkles and barcode lines in the lower third of the face is observed in the patient under examination.

**FIGURE 2:**
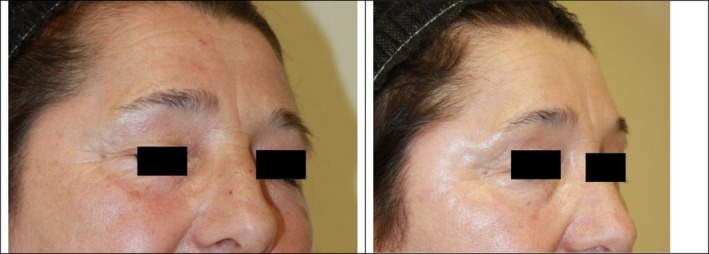
A reduction of eye wrinkles and an improvement in the texture and luminosity of the facial skin are noticed in the patient under examination.

**FIGURE 3:**
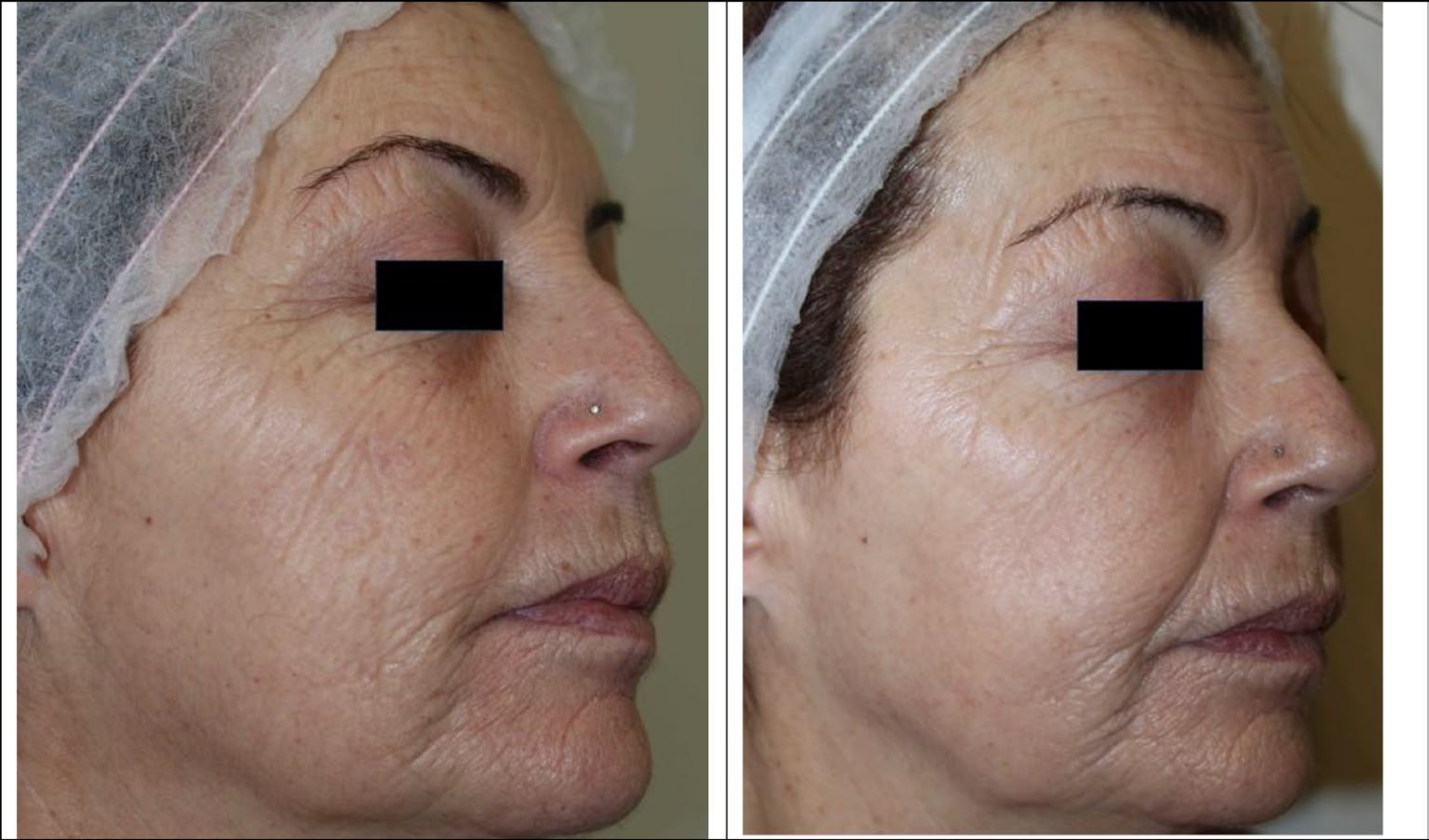
In this patient, a reduction of wrinkles, an improvement in the texture and an increased luminosity of the whole facial skin is observed.

**FIGURE 4:**
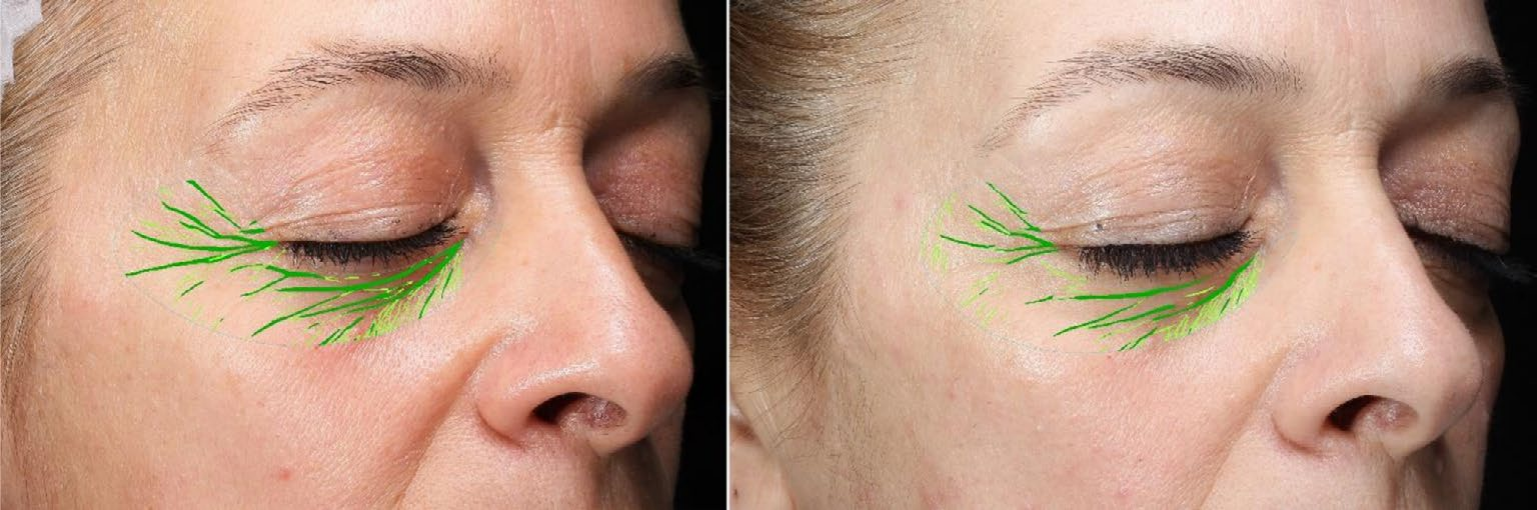
A facial image captured by Visia, showing a reduction of both deep (dark green) and superficial (light green) periocular wrinkles in a patient who carried out the protocol in the clinic.

Wrinkle severity in all three facial sections, particularly in the lower third, was reduced in most subjects, both when assessed with the “Wrinkle Assessment Scale” (Tables 1 and 2) and the WES (Tables 3 and 4). Both rating scales show clear and extremely similar improvements (29.13% improvement in one case and 27.51% in the other). Almost all patients, starting from higher severity levels, improved by one (Figures 1, 2) or even two degrees compared to the starting point (Figure 3), and only a few of the analyzed areas (42 out of 280 with the “Wrinkle Assessment Scale” scale and 12 out of 84 with the WES scale) remained unchanged. However, an improvement in texture was observed in these areas (Figure 4).

**FIGURE 5:**
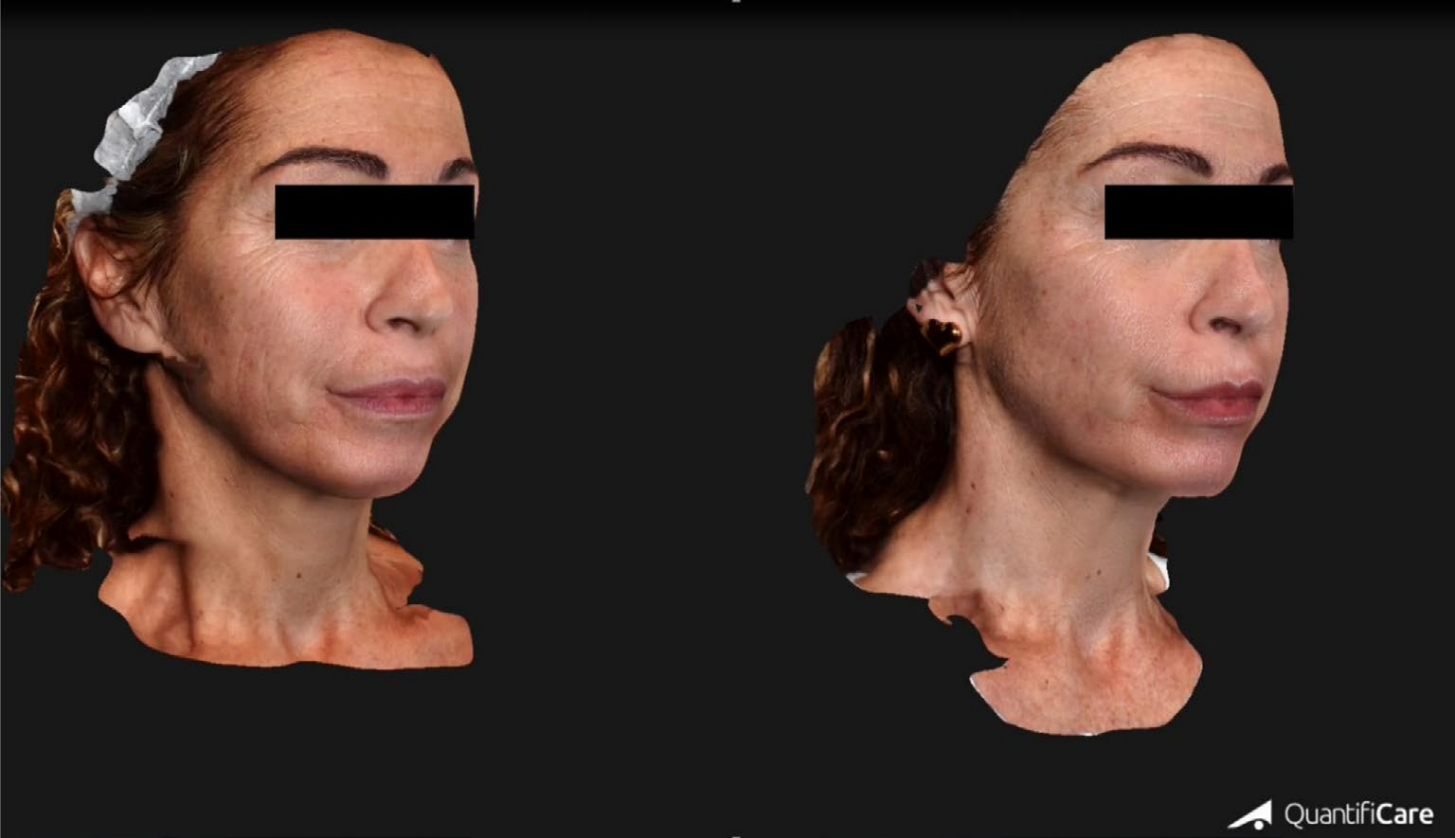
Comparison of the patient's face and décolleté with Quantificare before and after the cycle of treatments with VEMFtherapy.

We found that an aged appearance of the face compromises the quality of life, affecting social interactions and pejoratively defining the image individuals have of themselves. Appearance initial satisfaction score was 22.96, at the end of the treatment protocol it raised to an average of 26.82, showing a significant increase in patients' satisfaction with their appearance (Table 5). At the beginning of the protocol, most patients felt negatively affected by their age and assessed negatively their psychological well‐being in relation to their appearance, with an average score of 21.75. This assessment was then modified at the end of the treatment protocol to a value of 25.57, demonstrating how taking care of one's appearance and seeing it improved significantly impacts one's quality of life (Table 5). Finally, if at the beginning of the protocol most patients assessed their mirror image as being on average 3.4 years “older” than their biological age, at the end of the protocol this was significantly reduced to 1.36, demonstrating how the treatment has proved to be qualitatively and subjectively effective for patients who defined the look of their face as “(Table 5). The reduction of periocular wrinkles' absolute score measured with Visia provides a significant data about the overall effectiveness of the treatment (Figure 5). In conclusion, although the best approach to facial wrinkles is likely a combination of multiple therapies tailored to individual needs, our study highlighted the efficacy and safety of VEMFtherapy as a single therapy with a comprehensive approach to facial skin rejuvenation [27].

The three energies adopted have objectively demonstrated a synergistic effect in improving the degree of aging; a reduction in the depth and visibility of fine and deep wrinkles of all the areas of the face were observed, as well as an overall improvement of the skin to the touch and elasticity, also appreciated by patients. The parameter used for the grade of wrinkles severity and its improvement in all patients confirms that the treatment is effective in improving the appearance and degree of aging of the face; this ability is probably related to the remodeling of collagen and elastic fibers carried out thanks to the device as also demonstrated in other studies [36, 37].

We believe that part of the remodeling achieved, with reduced wrinkle depth, is due to the perpendicular repositioning of collagen fibers with respect to the stratum corneum, as demonstrated by Batista Castro et al. [39].

From the patient's subjective point of view, the Face‐Q parameter revealed both the significant impact on patients' interpersonal life quality of the appearance of the face and the judgment they had about their own look, and how the improvement of this aspect has consequently led to an improvement in patients' social and emotional lives, so much so that all the patients expressed satisfaction with the result obtained. While we documented a limited number of cases, we found consistent results across all patients, both in terms of wrinkle reduction and patient satisfaction.

## 5 Conclusions

The results obtained will certainly need to be confirmed by a larger sample of patients. Given this, we believe that, considering the improvement achieved, the total absence of side effects and downtime, and the high level of comfort expressed by patients, equal to 9 on a scale from 0 to 10, VEMFtherapy can become an important therapeutic option for skin aging.

## Author Contributions

The assignments in the conduct of this study were: performing treatments: Dr. Lombardo, Dr. Scacciati and Dr. Fabris; development of therapeutic protocol for each patient and supervision of the study: Dr. Menchini; writing: Dr. Lombardo.

## Funding

The authors have nothing to report.

## Conflicts of Interest

The authors declare no conflicts of interest.

## Supporting information


**Figure S1:** Classification of wrinkles—Menchini/Lombardo Scale.

## Data Availability

The data that support the findings of this study are available in the Bibliography section with DOIs 10.1111/jocd.70778.
