# Supplementary material for: Our Experience With VEMFtherapy on Skin Aging of Face and Neck
Source: J Cosmet Dermatol. 2026 Jul 19;25(7):e70778. doi: 10.1111/jocd.70778 (PMC13381811; doi:10.1111/jocd.70778)
Supplement: Supplementary file 1 — Figure S1: Classification of wrinkles—Menchini/Lombardo Scale. [file JOCD-25-e70778-s001.docx]

**Classification of wrinkles – Menchini/Lombardo Scale**

| Third of the face | I degree | II degree | III degree | IV degree | V degree |
| --- | --- | --- | --- | --- | --- |
| upper | 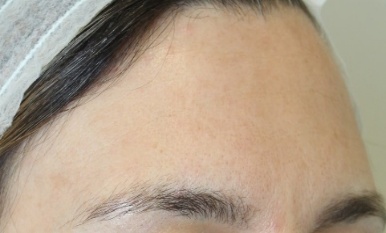 | 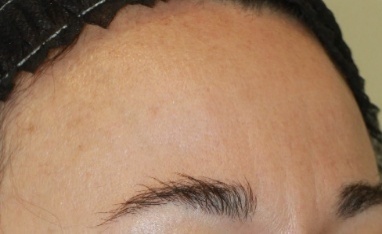 | 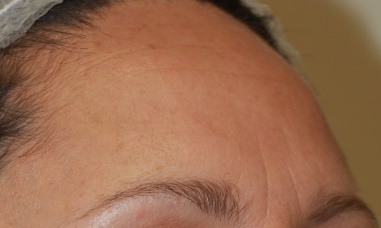 | 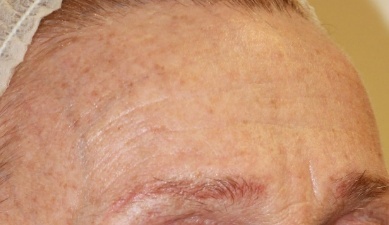 | 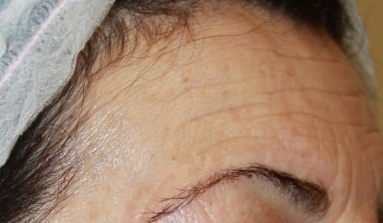 |
| middle | 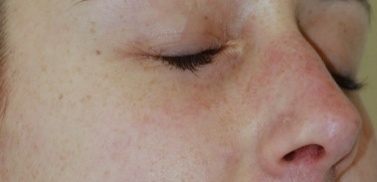 | 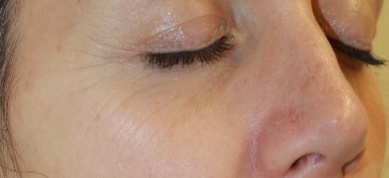 | 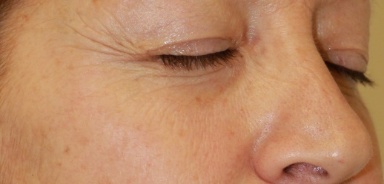 | 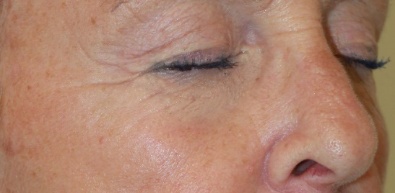 | 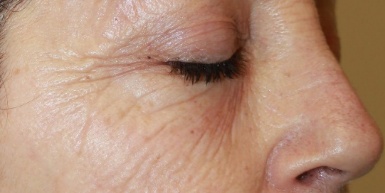 |
| lower | 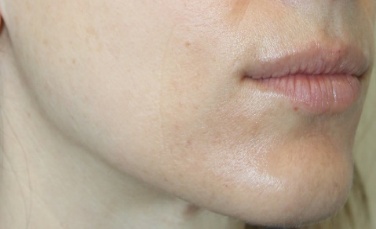 | 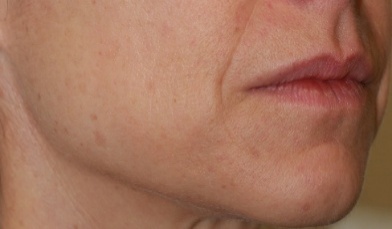 | 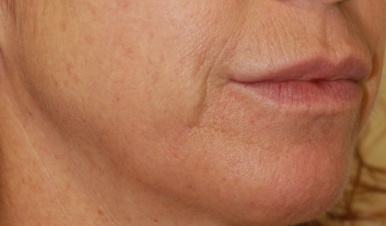 | 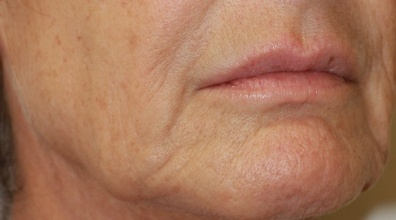 | 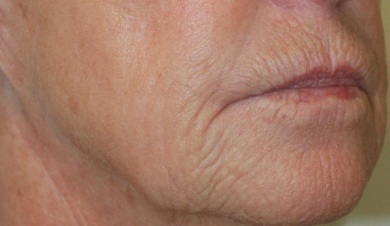 |

**Figure S1**
